# Ready When the Big One Comes? Natural Disasters and Mass Support for Preparedness Investment

**DOI:** 10.1007/s11109-021-09738-2

**Published:** 2021-08-21

**Authors:** Michael M. Bechtel, Massimo Mannino

**Affiliations:** 1 Department of Political Science, Washington University in St. Louis, St. Louis, MO 63130 United States; 2 Swiss Institute for International Economics and Applied Economic Research, University of St.Gallen, Bodanstrasse 8, 9000 St. Gallen, Switzerland

**Keywords:** Natural disasters, Policy myopia, Long-term policy problems, Voter preferences, Disaster policy, Affectedness, Misperceptions, Survey experiment

## Abstract

Societies can address collective threats such as natural disasters or pandemics by investing in preparedness (ex ante) or by offering compensation after an adverse event has occurred (ex post). What explains which of these options voters prefer? We study how personal exposure and policy knowledge affect mass support for long-term disaster preparedness, a type of long-term investment meant to cope with an increasingly destructive and frequent class of events. We first assess whether support for preparedness reflects personal affectedness and find that neither subjective nor geo-coded measures of disaster exposure predict policy preferences. Second, we explore whether this finding can be explained by misperceptions about the features of the available policy options. We find that revealing the damage reductions associated with preparedness systematically reduces opposition to long-term investment. These results suggest that opposition to preparing for collective threats may depend more on informational deficiencies than on personal experience with realized risks.

**Supplementary Information:**

The online version contains supplementary material available at 10.1007/s11109-021-09738-2.

## Introduction

Addressing major societal threats depends on the political will to back long-term investment in preparedness measures. Adapting to global warming and the associated increase in extreme weather events, for example, requires continuous investment today while most of the benefits will only be realized in the distant future.^1^ Similarly, preventive action that lowers societal and economic risks originating from pandemics, decaying public infrastructure, insolvent pension funds, and government overspending promises large, long-term gains. Because policymakers seek reelection, however, there exists an incentive to avoid the immediate, continuous costs of investing in preparedness. Instead, political elites may favor short-term responses that offer voters instant benefits even though this approach leaves societies underprepared for adverse events (Fox & Weelden, 2015) and forgoes the long-term returns to preventive and protective policy investment that will often be larger by an order of magnitude. Clearly, elections and the short time horizons they induce among policymakers offer one potential explanation for why countries underinvest in long-term policy problems. Yet, underpreparedness may also mirror opposition to such spending decisions among voters. Plausibly, if the public was willing to support the upfront investments required to pursue long-term reforms, governments would also be more likely to implement such policies. But what explains when voters are willing to back such long-term investments meant to address collective risks?

We explore this question by studying the mass politics of long-term investment in the context of natural disaster policy. Understanding the structure of voter preferences in this area of policymaking is important for several reasons. First, disaster policy has direct, real-world implications for large parts of the population since it is meant to help individuals cope with events that cause significant damage and may even trigger war-like postraumatic stress disorders (Weisaeth & Eitinger, 1993). Moreover, the frequency and intensity of extreme weather events has increased strongly over the past decades as have the associated human and economic losses.^2^ Second, we are able to evaluate several theoretical accounts of voter preferences over long-term policy investment. A prominent and intuitively appealing argument in this literature holds that personal affectedness shapes political attitudes. By testing the impact of personal exposure to natural disasters—an event that is much more intense and threatening than the experiences voters have in their daily interactions—on public spending preferences we consider a most-likely case that probes the limits of affectedness-based theories of opinion formation. Third, since disaster preparedness promises considerable efficiency gains over the provision of relief aid (Healy & Malhotra, 2009), understanding the conditions under which voters are willing to back long-term adaptation strategies as opposed to short-term fixes is also of significance from a public spending perspective.

Coping with natural disasters requires a policy that sets out the extent to which public resources will be invested in prevention or compensation. Preventive measures necessitate long-term investment in damage-reducing infrastructure (ex ante) whereas compensation means to provide relief aid in the aftermath of a natural disaster to offset experienced losses (ex post). An influential argument in the existing literature posits that disaster policy preferences are affected by personal exposure to extreme weather events. According to the “learning-through-experience” model, personal affectedness sets in motion a cognitive process in which individuals update their deflated beliefs about the likelihood and severity of extreme weather events which causes them to revise their policy views.

Consistent with this expectation, several studies have demonstrated that weather fluctuations affect climate warming beliefs in the US (Egan & Mullin, 2012; Howe et al., 2019a) and there also exists some evidence suggesting that self-reported flood risk is associated with a higher willingness to consider relocation (de Koning et al., 2019). Others, however, have questioned whether environmental conditions systematically affect policy preferences. For example, previous tsunami exposure correlates positively with municipality-level damage which could indicate that the public may fail to learn from past disaster experience (Plümper et al., 2017). Indeed, recent survey evidence suggests that exposure to extreme weather events is not associated with higher climate concern (Bergquist & Warshaw, 2018) and a review of previous studies even identifies a “risk perception paradox” since disaster exposure may lead individuals to deem it less likely to be affected by extreme weather events in the future (Wachinger et al., 2013). Given these and other mixed results, Howe et al. (2019b) advise future work to more carefully distinguish between self-reported and objective measures of personal exposure and be mindful of causal identification issues.

We extend this literature by studying the relationship between personal exposure to natural disasters and support for preparedness investment. This analysis allows us to explore an implication of the learning-through-exposure argument which suggests that disaster exposure leads individuals to revise their assessments of the frequency and intensity of natural disasters. These revised assessments of the underlying risk due to experiential learning should lead individuals to change their preferred policy responses. We find, however, that being personally affected by extreme weather events is not systematically correlated with policy preferences. This result holds irrespective of whether we measure personal exposure based on respondents’ self-assessed disaster affectedness or geo-coded information about whether a respondent lives in a region that has been affected by extreme weather events in the past. The evidence also indicates that respondents who deem themselves more likely to be affected by natural disasters in the future are not significantly more willing to back long-term disaster preparedness investment. Instead, our estimates reveal that a general measure of respondents’ patience is a robust predictor of policy preferences. Individuals who are more patient are more willing to invest in preparedness (instead of relief aid) and are more supportive of improving local protective infrastructure that reduces disaster damage.

Second, we examine a potential explanation for the absence of a systematic relationship between personal exposure and policy preferences: even though voters may be adversely affected by a disaster and revise their beliefs about the intensity and frequency of extreme weather events, this event could fail to correct their comparative assessments of the available policy options, i.e., the higher perceived effectiveness of disaster relief as opposed to preparedness investment. In other words, the lack of a systematic relationship between personal affectedness and voter preferences may reflect misperceptions about the economic features of preventive vs. reactive policy. Specifically, voters could prefer the ex post response, i.e., providing relief aid in the aftermath of natural disasters, because of two types of policy-related misperceptions: underestimating the effectiveness of long-term investment in damage-preventing infrastructure and overestimating its costs.

Testing this argument is challenging with observational data. Therefore, we devise two survey experiments that randomize information about the impact and costs of long-term investment in damage prevention to explore the causal drivers of support for disaster preparedness. The results suggest that both types of information—policy effectiveness and costs—systematically affect support for long-term investment in the expected direction. In contrast to previous research that has documented pronounced partisan divides in both climate opinions (Jenkins-Smith et al., 2020) and disaster-related behavior (Long et al., 2020), we find that the effects of these policy features are quite comparable across respondents’ partisan attachments. Consistent with our correlational findings, the results also suggest that the policy preferences of those with higher exposure to natural disasters do respond equally to revealing the economic advantages of long-term disaster policy. Taken together, our findings are consistent with the view that policy features may be more important for explaining the willingness to back long-term investment to address large-scale problems than personal experience with the underlying risks.

## Natural Disasters and Long-Term Investment

Democracies incentivize incumbents to provide welfare-enhancing policies in exchange for voter support in free, recurring elections (Downs, 1957). This increases the tendency of policymakers to provide public instead of private goods, but their policy choices run the risk of reflecting the temporal incentives set by short-term re-election concerns and the time horizons that myopic voters employ when forming retrospective evaluations of incumbent performance (Achen & Bartels, 2018; Heersink et al., 2017; Healy & Malhotra, 2013; Jacobs, 2011). As a consequence, governments could prefer providing disaster relief because this offers voters immediate, visible, and concentrated benefits instead of investing in disaster preparedness despite the long-term efficiency gains that this investment may generate. The available evidence is consistent with the prediction that policymakers massively under-invest in preparedness. According to data from the Consolidated Federal Funds Report (1985 to 2010), federal authorities spent about thirty times as much tax dollars on the provision of disaster relief than on preparedness investment.^3^ While such myopic policymaking could result from politicians’ short time horizons which are primarily driven by re-election concerns, underpreparedness could also mirror voter preferences. In what follows we develop several theoretical arguments that may help explain mass support for disaster preparedness.

Political economy theories argue that individuals will support policies that they expect to benefit from personally. Existing research suggests that these expectations strongly depend on personal exposure and vulnerability. Having been affected by recent weather shifts changes beliefs about global warming (Egan & Mullin, 2012) and increases support for both climate action as well as political candidates who propose progressive environmental policies (Rudman et al., 2013). These findings are consistent with previous work on the impact of environmental events on subjective risk assessments (Weisaeth & Eitinger, 1993) and the strong correlation between perceived and actual mortality risks for a large set of life-threatening events (Friedman, 2019). Specifically, personal exposure to natural disasters could set in motion a learning process in which individuals revise their preferences over which allocation of public resources they deem most beneficial. This assessment may include both: the level of preventive spending, e.g., investment in damage-reducing infrastructure such as flood control dams (Lowry, 2003), and the proportion of resources devoted to preventive or protective measures (ex ante) as opposed to compensatory spending (ex post).

The learning-through-experience argument suggests that those who have had personal experience with natural disasters in the past and those who have not differ markedly in their beliefs about disaster risks and their impacts. Exposure to extreme weather events should increase expectations about the probability of natural disasters and the likelihood of being affected in the future. This type of experiential learning will shift voters preferred policy options. If an individual deems the risk of being affected by extreme weather events to be quite low, investing tax dollars in damage-preventing measures will seem unattractive. Instead, public resources devoted to the provision of disaster relief may appear preferable as they provide immediate benefits to those in need. In contrast, those who have been affected by natural disasters should be more likely to believe that natural disasters will occur in the future and therefore expect preparedness spending to be a useful investment. This means that support for preparedness spending should be higher among those who have been affected by natural disasters in the past and those who think it is more likely that they will be affected in the future.

In addition, disaster exposure could increase the likelihood of recognizing the relative costliness of providing relief aid to compensate for experienced losses. This, in turn, would increase the attractiveness of preventive as opposed to compensatory spending. If the experiential learning argument is valid, one would expect individuals who have been affected by a natural disaster to prefer allocating a larger share of public resources into preparedness instead of relief spending. We note that this prediction relies on an informational mechanism that affects individual assessments of the relative cost-effectiveness of preparedness versus relief spending.^4^ We offer a more direct assessment of this mechanism when analyzing our experimental data.

If policy preferences reflect individuals’ assessments of the expected profitability of investing public resources in damage-reducing measures, support for improving disaster preparedness should also depend on general beliefs about the existence of environmental problems and the idea that humans can take steps to address them. This type of environmentalism comprises cognitive, affective and conative dimensions (Maloney & Ward, 1973). Voters who think that environmental problems exist (cognitive component), perceive them as a threat (affective component), and would like to see policy action (conative component) will generally exhibit a higher willingness to invest in damage-reducing preparedness measures.

Disaster preparedness requires investing resources today in order to benefit from reduced disaster damage in the future (Jacobs & Matthews, 2015) which means that disaster policy choice constitutes a delayed investment problem. This suggests that both individual time horizons may help explain disaster policy preferences. Previous research has shown that time preferences are significant predictors of the willingness to incur the costs of more energy-efficient products (Newell & Siikamäki, 2015) and correlate positively with support for military interventions which often require long-term deployment of troops in the face of repeated setbacks (Kertzer, 2017). According to this view, more patient voters will discount the future benefits of damage-reducing infrastructure less than impatient individuals. If this reasoning is valid, patient voters should be more willing to back long-term investment in disaster preparedness than individuals with shorter time horizons.

Disasters occur probabilistically. Consequently, the returns to preparedness investment are associated with uncertainty as they will only be realized when a natural disaster happens. Whether voters consider investing in preventative measures preferable and potentially more important than providing relief aid may therefore reflect a divide between more and less risk averse individuals. Voters who are more willing to accept uncertainty in general should be less likely to back preparedness spending than individuals who tend to be risk averse.

Arguably,  even if voters had relatively similar subjective assessments of risks such as those originating from extreme weather events, the willingness to support costly policy action may vary because of differences in partisanship. Partisan leanings could be especially important when forming policy views on long-term investment as this involves the use of tax money and state intervention, both of which are closely related to ideological beliefs about the appropriate role of government. While Democrats generally prefer higher levels of state intervention, Republicans embrace the idea of lean government paired with greater personal responsibility (Feldman & Zaller, 1992). If voters draw on their partisan orientation when forming preferences over disaster policy this argument predicts that left-leaning respondents should be more willing to support long-term investment than those on the ideological right.

Lastly, support for disaster preparedness may also reflect beliefs about the credibility of the policy promises made by elected officials (Jacobs & Matthews, 2015). Voters with higher levels of political trust may deem it more likely that the incumbent will provide sufficient relief in the aftermath of a natural disaster. This argument would predict that political trust should correlate negatively with support for preparedness spending.

## Data and Measurement

We evaluate our theoretical predictions using data from an online survey that we fielded to a quota sample of 2,618 American citizens (Bechtel & Mannino, 2021).^5^ Online Appendix Table A.1 reports the distribution of sociodemographic characteristics in the survey sample and compares it to the target population of American citizens. Overall, the sample closely matches the socio-demographic composition of the target population. All our analyses use weights but the results remain substantively unchanged when we analyze the unweighted data.

### Measuring Preferences for Long-term Investment: Damage Prevention vs. Compensation

We use two complementary survey items to capture public opinion on disaster policy. Our first dependent variable *Preparedness Investment* is based on a novel question that asks respondents to distribute a fixed budget between improving disaster preparedness and the provision of disaster relief:

Suppose you had to decide how much of a $100 million budget is being spent on preparing for disasters and on providing disaster relief, how would you divide up the money? Please use the bars below to indicate how much of a $100 million budget you would spend on preparing for disasters and how much you would spend on providing disaster relief. Please note that you cannot spend more than $100 million in total.

Our dependent variable records the amount that respondents allocate to improving long-term disaster preparedness which can range from $0 to $100 million. We note that the underlying question intentionally requires individuals to make a choice that entails a trade-off. This design improves over items in which participants are allowed to express their policy views in a setting in which there are no costs to assigning policy or spending priorities. Second, by asking individuals to make a financial allocation decision under budget constraints, the responses promise to be more directly comparable across individuals as they are less dependent on subjective understandings of answer scales that attempt to capture policy importance, concern, or approval.

Our first item measures preferences for long-term investment in disaster preparedness as opposed to relief spending in general. We complement this dependent variable with a second item that elicits support for public investment in damage prevention in the context of a local flood control dam scenario. Albeit it simplifies many of the aspects associated with the potential environmental and social consequences of flood control dams, this survey item should still allow us to capture variation in support for this type of preparedness investment. Also, it is closely tied to empirically relevant scenarios: floods have proven to be extremely destructive natural disasters with worldwide economic losses estimated at over $600 billion in the 1995–2015 period (CRED, EM-DAT and UNISDR, 2017), p. 24.^6^ Flood control dams are an important example of an investment in a region’s damage-preventing infrastructure. The flood dam question was:

Now we would like to know what you think about the following proposal: Your local government worries that periods of heavy rainfall could cause the river that flows through the city to flood in the future, thereby causing severe economic damage. For this reason, it proposes the construction of a dam that could reduce the damage caused by a flood. The building of a dam would cost $[2, 8, 14] million and would reduce future damage by about $20 million because the cost of rebuilding the bridge would decrease from $100 to $80 million. Some say that it is not worth building the dam because the river only floods once every ten years. If you could vote on this policy proposal in a referendum, how likely is it that you would vote in favor or against it?

We randomized the information in parentheses treating the $2 million treatment as the control condition and report the results further below. We code answers such that they measure support for the flood control dam from 1 (strongly oppose) to 10 (strongly support) and refer to this measure as *Dam Support*. We note that the flood control dam item makes the intertemporal trade-off between investing today and reducing future damage explicit: building a dam to prevent river flooding is associated with immediate costs but promises delayed gains in the form of reduced future losses.

### Predictors

We use several variables to capture respondents’ self-reported as well as objective exposure to natural disasters. Our first variable *Affectedness* is based on a question that asks respondents to indicate whether they or any of their household members have been affected by a natural disaster (e.g., tornadoes, floods, earthquakes, droughts, or other adverse weather events) in the past ten years and how (e.g., closed roads, shutdowns of schools, restricted access to food or water, evacuation, injury, financial losses). Absent theoretical reasons for why these types of personal experience should matter differently when analyzing disaster policy preferences, we use respondents’ self-reported exposure to create an additive affectedness index. We standardize this affectedness index such that it can range from 0 (not at all affected) to 1 (strongly affected). Online Appendix Table A.1 (p. 3) reports the exact question wording along with descriptive statistics. We also measure individuals’ beliefs about whether the county they live in will be affected by extreme weather events in the future and generate the corresponding variable *Disaster Risk Beliefs* which ranges from 1 (very unlikely) to 10 (very likely).

These measures rely on respondents’ self-assessments. To generate objective indicators of affectedness we use geographical information about a respondent’s place of residence which we collected at the county-level. We match this data with geo-coded information about natural disaster occurrence and damage in the United States from Bechtel and Mannino (2021) to create two variables.^7^*Injuries* counts the number of individuals that have been injured by natural disasters and *Economic Damage* measures the associated financial losses. We account for differences in individuals’ time horizons and risk aversion in our estimations using the standard survey questions. In addition, we measure respondents’ political trust and include a survey item that has been used in previous research to generate an individual-level measure of environmentalism (Bechtel & Scheve, 2013). Online Appendix Table A.3 (p. 4) reports the exact question wording along with descriptive statistics.

## Explaining Mass Support for Disaster Preparedness

**Fig. 1:**
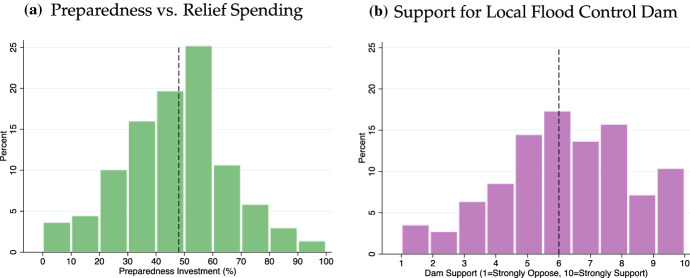
Preferences for preparedness investment and support for flood control dam. *Note*: Panel (a) shows respondents’ preferred long-term investment choices. Panel (b) shows the distribution of support for building a local flood control dam. Dashed vertical lines indicate the median response. Results are based on respondents’ answers in the experimental control conditions (see below for details). Preparedness Spending N=873, Dam Support N = 874

How strongly do voters like to invest in preventive as opposed compensatory disaster policy? The left panel in Fig. 1 shows respondents’ preferred spending allocations which reveal considerable variation in disaster policy preferences. The vertical line indicates the median response. We find that the median individual allocates about 48% to improving disaster preparedness and the corresponding 95 percent confidence interval excludes 50. This suggests that the public prefers an investment level that is significantly below 50%.^8^ The right panel in Fig. 1 shows the distribution of support for the local flood control dam. We again find a high degree of variation in respondents’ answers although a majority expresses levels of support that exceed the midpoint of the 1 to 10 scale. At the same time, more than 30% of all respondents either oppose investing in a local flood control dam or are indifferent. It is worth noting that the dam support measure did not confront respondents with a trade-off in which resources spent on one policy option would reduce the amount available for another. Therefore, supporting the dam was not associated with any obvious opportunity costs which may have inflated levels of support. It is all the more striking that a non-trivial number of respondents decided not to back this type of long-term investment. In what follows we test whether variables related to the theoretical considerations above help explain variation in individuals’ disaster policy preferences.

### Personal Affectedness: The Role of Experiential Learning and Risk Assessments

What explains the willingness to support long-term investment in damage-preventing measures? We first investigate whether personal exposure to extreme weather events matters by regressing our two outcome variables on measures that capture personal disaster exposure along with a full set of sociodemographic control variables (income, age, education, gender, and race).^9^

Models 1 to 6 in Table 1 report the results for the *Preparedness Investment* outcome variable and Models 7 to 12 report the estimated coefficients for the *Dam Support* variable which we multiply by 10 so that both outcome measures have roughly the same range. We first evaluate the importance of personal disaster exposure in explaining support for long-term preparedness investment. We convert the self-reported affectedness measure into three binary indicator variables based on the terciles of the observable distribution. Model 1 suggests that compared to respondents with low affectedness (the reference group) neither individuals with medium nor high levels of disaster exposure are more supportive of preparedness investment. Furthermore, the results in Model 7 in Table 1 suggest that personal disaster exposure also fails to predict support for building a local flood control dam. These results are unchanged when we re-estimate Models 6 and 12 using a binary affectedness indicator that distinguishes between no reported disaster exposure and having reported at least some level of affectedness (Online Appendix Table A.5). These results question the empirical validity of the experiential learning argument.

To account for subjective expectations about the likelihood of disaster affectedness Models 1 and 7 include binary indicators that are based on the terciles of the *Disaster Risk* variable. We find that subjective risk evaluations fail to systematically correlate with individuals’ preparedness spending preferences. Although respondents with higher subjective risk beliefs are significantly more likely to support investment in a local flood control dam (Model 7), the results reported in Model 11 of Table 1 suggest that this finding is not robust to controlling for political factors related to general levels of environmentalism, partisanship, and trust.

**Table 1:** Personal Affectedness and Support for preparedness

Outcome:	Preparedness Investment (%)						Dam Support (1–10) × 10					
Model:	(1)	(2)	(3)	(4)	(5)	(6)	(7)	(8)	(9)	(10)	(11)	(12)
*Self-reported exposure*												
Affectedness: Medium	0.69				0.46		1.55				0.74	
	(0.89)				(0.99)		(1.09)				(1.18)	
Affectedness: High	0.60				− 0.03		− 1.35				− 2.16	
	(0.98)				(1.12)		(1.29)				(1.43)	
Disaster Risk Beliefs: Medium	0.10				− 0.37		− 0.73				− 0.62	
	(0.91)				(0.99)		(1.14)				(1.23)	
Disaster Risk Beliefs: High	0.26				− 0.20		2.67**				2.06	
	(0.98)				(1.08)		(1.22)				(1.33)	
*Geo-coded exposure*												
Economic Damage: Medium		0.84				1.39		0.75				0.84
		(0.93)				(1.09)		(1.14)				(1.32)
Economic Damage: High		0.19				− 0.03		0.08				− 0.11
		(1.09)				(1.28)		(1.45)				(1.63)
Injuries: Medium		− 0.79				− 0.84		1.13				0.61
		(0.89)				(1.02)		(1.14)				(1.30)
Injuries: High		− 0.87				− 0.68		− 0.95				− 2.07
		(1.10)				(1.28)		(1.39)				(1.58)
Patience: Medium			0.79		0.60	0.91			1.05		0.92	0.66
			(1.07)		(1.09)	(1.08)			(1.33)		(1.35)	(1.34)
Patience: High			2.09*		2.30**	2.42**			6.73***		6.80***	6.35***
			(1.09)		(1.12)	(1.11)			(1.40)		(1.42)	(1.41)
Risk Acceptance: Medium			1.04		0.73	0.88			0.67		0.26	0.58
			(1.37)		(1.41)	(1.37)			(1.72)		(1.73)	(1.73)
Risk Acceptance: High			0.94		0.39	0.51			1.80		1.60	1.80
			(1.45)		(1.48)	(1.45)			(1.83)		(1.87)	(1.85)
Environmentalism: Medium				1.25	1.36	1.40				3.16***	3.23***	3.06**
				(0.89)	(1.01)	(0.99)				(1.10)	(1.21)	(1.20)
Environmentalism: High				3.17***	4.11***	4.10***				− 1.03	− 0.94	− 1.18
				(1.04)	(1.20)	(1.14)				(1.26)	(1.41)	(1.40)
Responsibility Beliefs: Medium				1.95*	1.61	1.75				0.31	0.40	1.03
				(1.04)	(1.17)	(1.15)				(1.32)	(1.47)	(1.46)
Responsibility Beliefs: High				2.05**	1.77	2.15*				0.22	0.38	0.76
				(1.01)	(1.13)	(1.10)				(1.26)	(1.40)	(1.39)
Political Trust: Medium				− 1.04	− 1.06	− 1.04				− 0.93	− 0.79	− 0.55
				(1.03)	(1.16)	(1.15)				(1.27)	(1.43)	(1.43)
Political Trust: High				− 0.71	− 0.20	− 0.41				0.59	− 0.07	0.17
				(1.13)	(1.26)	(1.25)				(1.43)	(1.59)	(1.58)
Partisanship: Republican			0.08	0.05	0.05	0.51			− 5.57***	− 4.08***	− 5.36***	− 4.89***
			(1.12)	(1.08)	(1.20)	(1.18)			(1.49)	(1.40)	(1.56)	(1.56)
Partisanship: Other			− 0.13	0.09	− 0.04	0.16			− 5.73***	− 5.90***	− 5.57***	− 5.41***
			(0.94)	(0.86)	(0.97)	(0.96)			(1.17)	(1.10)	(1.21)	(1.21)
Income: Medium	− 0.69	− 0.39	− 0.80	− 0.52	− 0.55	− 0.38	1.93*	2.97**	1.77	1.88	1.22	1.98
	(0.95)	(0.95)	(1.01)	(0.93)	(1.03)	(1.01)	(1.17)	(1.18)	(1.26)	(1.16)	(1.27)	(1.28)
Income: High	0.57	0.81	0.04	0.54	0.37	0.29	2.74**	3.60***	2.72**	2.83**	1.94	2.67**
	(1.01)	(1.01)	(1.08)	(0.99)	(1.10)	(1.10)	(1.25)	(1.24)	(1.30)	(1.21)	(1.33)	(1.32)
Age: 25–44	− 0.42	0.06	0.20	0.03	− 0.86	− 0.12	− 3.84**	− 4.67***	− 4.88***	− 4.49***	− 4.18***	− 4.99***
	(1.19)	(1.21)	(1.29)	(1.18)	(1.31)	(1.31)	(1.51)	(1.50)	(1.59)	(1.48)	(1.62)	(1.59)
Age: 45–64	− 2.75**	− 2.34*	− 2.14	− 1.75	− 1.91	− 1.48	0.09	− 0.88	− 0.97	− 0.86	− 0.97	− 1.58
	(1.23)	(1.22)	(1.33)	(1.21)	(1.38)	(1.36)	(1.52)	(1.49)	(1.62)	(1.50)	(1.68)	(1.65)
Age: 65 or more	− 4.17***	− 3.77***	− 3.67**	− 2.84**	− 3.26**	− 2.83*	1.10	0.38	0.71	− 0.13	0.56	− 0.08
	(1.38)	(1.36)	(1.49)	(1.38)	(1.57)	(1.53)	(1.73)	(1.67)	(1.80)	(1.70)	(1.88)	(1.84)
Education: High School	− 1.20	− 1.03	− 1.20	− 0.80	− 1.57	− 1.24	2.21	1.36	1.55	1.18	2.26	1.17
	(1.43)	(1.41)	(1.49)	(1.39)	(1.55)	(1.51)	(1.80)	(1.77)	(1.88)	(1.73)	(1.92)	(1.89)
Education: Some College	− 0.81	− 0.70	− 0.69	− 0.26	− 1.20	− 0.98	1.71	0.50	− 0.33	0.14	0.24	− 1.02
	(1.44)	(1.42)	(1.53)	(1.40)	(1.59)	(1.55)	(1.83)	(1.80)	(1.93)	(1.76)	(1.97)	(1.93)
Education: BA	0.76	1.38	1.08	1.41	0.50	0.95	2.23	1.15	0.51	0.55	1.52	0.04
	(1.55)	(1.53)	(1.64)	(1.52)	(1.71)	(1.67)	(2.01)	(1.99)	(2.12)	(1.95)	(2.15)	(2.13)
Education: Advanced Degree	3.65**	3.38*	3.35*	3.72**	2.77	2.73	2.56	1.44	1.46	0.56	2.37	1.24
	(1.75)	(1.76)	(1.87)	(1.73)	(1.91)	(1.90)	(2.34)	(2.35)	(2.50)	(2.28)	(2.54)	(2.52)
Female	− 1.24	− 1.24	− 0.75	− 1.32	− 0.78	− 0.81	− 0.12	0.16	− 0.07	0.01	− 0.25	− 0.15
	(0.83)	(0.83)	(0.89)	(0.81)	(0.90)	(0.90)	(1.00)	(1.01)	(1.06)	(0.98)	(1.07)	(1.08)
Race: African American	1.09	1.46	1.35	0.83	0.33	0.96	− 2.34	− 2.40	− 3.44*	− 3.65*	− 2.93	− 2.82
	(1.47)	(1.44)	(1.52)	(1.42)	(1.57)	(1.52)	(1.89)	(1.87)	(1.97)	(1.87)	(2.02)	(2.00)
Race: Hispanic	1.42	0.92	0.94	0.61	0.69	0.61	0.96	0.83	0.10	0.73	0.78	0.85
	(1.60)	(1.61)	(1.69)	(1.56)	(1.69)	(1.70)	(2.11)	(2.08)	(2.23)	(2.02)	(2.25)	(2.24)
Race: Other	1.49	0.97	0.24	1.03	0.40	0.05	− 3.52*	− 3.68**	− 4.11**	− 4.00**	− 3.47*	− 3.48*
	(1.40)	(1.44)	(1.53)	(1.40)	(1.50)	(1.53)	(1.87)	(1.87)	(2.03)	(1.85)	(2.05)	(2.03)
Constant	48.06***	47.99***	50.14***	45.14***	48.46***	48.68***	62.48***	63.73***	71.01***	68.08***	69.96***	62.32***
	(1.62)	(1.50)	(3.99)	(2.05)	(4.25)	(3.90)	(2.03)	(1.86)	(4.61)	(2.54)	(4.89)	(5.45)
State Fixed Effects					Yes	Yes					Yes	Yes
Observations	2,517	2,543	2,271	2,618	2,192	2,241	2,517	2,543	2,271	2,618	2,192	2,241
R-squared	0.021	0.019	0.031	0.025	0.044	0.042	0.020	0.017	0.061	0.034	0.075	0.069

OLS coefficients shown with robust standard errors in parentheses (***$p<0.01$, **$p<0.05$, *$p<0.1$)

So far the results rely on self-reported measures of disaster affectedness and risk assessments. The potential limitations of such measures, for example, imperfect recall and social desirability bias, and the endogeneity problems associated with observational data are well known. To begin addressing these issues, we employ our geo-coded affectedness measures of disaster exposure that record economic damage and injuries. The results reported in Models 2 and 8 in Table 1 suggest, however, that these variables are not systematically related to disaster policy preferences. The combined evidence based on self-reported and geo-coded measures of affectedness indicates that personal exposure—in spite of its theoretical importance—offers surprisingly little insight into the mass politics of disaster preparedness investment. We elaborate on the implications of this finding for our understanding of the politics of long-term investment and the ability of democracies to adopt reforms that overcome myopic policymaking in the conclusion.

### Risk Aversion, Patience, and Environmentalism

Improving disaster preparedness requires the willingness to make investments today in order to realize benefits in the future that are uncertain. Do individuals’ general time horizons and risk aversion help predict the willingness to invest in preparedness measures? The results suggest that more risk-acceptant respondents are not significantly more willing to back long-term investment. However, we find that more patient individuals prefer significantly higher levels of preparedness spending. Consistent with this result, the estimates also suggest that patient respondents are also significantly more supportive of building a local flood control dam (Table 1, Models 9, 11, and 12).

The results in Models 5 and 6 suggest that environmentalists devote significantly more resources to long-term disaster policy investment. For our dam support measure, it is the moderate environmentalists who tend to be more in favor of local preparedness investment while the most environmentalist respondents are indistinguishable from the reference group of individuals with low levels of environmentalism. This non-linear pattern indicates that the role of environmentalism varies depending on whether we explore preferences over general disaster policy or a local investment project. A potential explanation is that, in contrast to general preferences over the relative importance of preparedness as opposed to relief spending, support for a flood control dam is equivalent to backing a specific, invasive infrastructure that may have a negative impact on biodiversity and the goal of preserving natural landscape for future generations. This could reduce the willingness of those who are particularly environmentally conscious to back investing in preparedness projects.

Most of the political variables do not systematically correlate with disaster policy preferences. An exception is that those who identify with the Republican party are significantly less likely to support a local flood control dam than Democratic respondents. This could reflect that Republicans view support for investing in protective infrastructure such as a flood control dam as a decision about whether to increase the overall level of public spending. Therefore, opposition to damage-preventing investment could mirror that Republicans prefer small government. Lastly, we re-estimate all specifications using a tobit model to account for the boundedness of our outcome variables. As Online Appendix Table A.6 shows, the results remain unchanged.

Taken together, the results suggest that neither subjective nor objective measures of disaster exposure offer insights into the formation of policy preferences. One explanation for this finding is that voters misperceive the relative attractiveness of preventive as opposed to compensatory policy instruments and personal exposure fails to offer the information needed to revise those policy beliefs. Testing the misperception argument is difficult using correlational data. Therefore, we devise two experiments that reveal information about the available policy options to test whether misperceptions can help explain the lack of a systematic relationship between disaster exposure and policy preferences. 

## Disaster Policy Preferences and Misperceptions About Preparedness

A large literature on voter sophistication has documented that citizens tend to possess little knowledge about basic policy questions (Delli & Keeter, 1996; Nadeau et al., 1993). It seems even less likely that individuals are well informed about the economic returns to investing in damage-preventing measures relative to compensating for realized losses by providing relief aid. The lack of information about the relative advantages and disadvantages of the available policy options is further amplified if the underlying problem concerns a temporally distant issue which is associated with additional uncertainty (Jacobs & Matthews, 2012). This suggests that support for long-term investment may be sensitive to factors that affect the uncertainty surrounding the costs and benefits of long-term spending decisions. Policy uncertainty could limit both the ability of societies and the willingness of policymakers to draw lessons from natural disasters that lead to policy change (Birkland, 2006). In addition, these two factors may interact. For example, Jacobs and Matthews (2015) show that the trustworthiness of state actors responsible for implementing long-term reforms affects voter support since individuals may discount their policy promises.

If voters have limited knowledge about the precise features of a specific policy choice, they may generally lean towards supporting the less risky, more immediate, and already known option of providing disaster relief. If this reasoning is valid, information about the economic advantages of investing in disaster preparedness should lead individuals to reassess the attractiveness of this policy option, causing them to shift their opinions in favor of long-term investment. In other words, making the effectiveness of preparedness transparent should weaken opposition to investing in preventive measures relative to the short-term option of providing relief aid. While the long-term effectiveness of preparedness should increase support for such measures, opinion formation may also be sensitive to the immediate, relative costs of preparedness compared to the provision of relief aid. Specifically, voters should offer stronger support for preparedness spending if the costs of these measures decrease or, alternatively, the efficiency of the preventive policy option is revealed. These theoretical predictions can reflect several potential mechanisms which we elaborate on and test further below.

### Long-term Disaster Policy Experiment: Design

Our first experiment evaluates whether support for disaster preparedness may be explained by misperceptions or a lack of information about the economic features of the underlying policy option. Certainly, we would not expect this type of policy information to be the sole driver of policy preferences or to be able to increase support for long-term policy to the theoretical maximum or the social optimum. Instead, our goal is to test whether the misperceptions argument finds empirical support by experimentally revealing information about the features of the long-term policy option that “corrects” individuals’ baseline beliefs. We implement this experiment by  providing all respondents with the same introduction and outcome question (see above), while varying the main body of the text. The exact wording of the introduction was as follows:

Natural disasters such as floods, fires, earthquakes, tornadoes, or windstorms affect thousands of people every year and have already caused more than $1 trillion in economic damage since the 1980s.

We then randomly assigned individuals to each of three experimental conditions. The first group received the introduction without any additional information. The second group in addition received information about the benefits that long-term policy investment would provide which we expressed in terms of damage reductions:

Experts say that preparing for natural disasters strongly reduces future economic damage caused by natural disasters. For example, the cost of Hurricane Katrina could have been reduced from $100 billion to $7 billion if the government had invested more in disaster preparedness measures. In other words, investing in disaster preparedness is much less costly than providing disaster relief.

While other types of information treatments seem feasible,^10^ the setting and figures used in the *Damage Reduction* condition were based on an important real-world example.^11^ In the control group, we provided no additional information about the features of the long-term investment option. A potential criticism is that providing no information about the policy options is unrealistic because respondents may be unaware of the ways in which societies could address disaster risks. Therefore, any differences in spending preferences could either result from not knowing which policy options exist or misperceptions about their relative economic features. To address this concern we added an alternative control condition in which one third of our respondents received information about the ex post option of compensating disaster losses (*Compensation* condition):

To deal with the consequences of natural disasters, the US government can invest in disaster relief measures such as providing medical assistance, sending technical experts, or offering financial support.

Our outcome variable *Long-Term Investment*—which we already introduced above—asked respondents to decide about how to allocate a $100 million budget between disaster preparedness and disaster relief.

### Disaster Policy Experiment: Results

Table 2 reports the results as estimated within a linear regression model.^12^ We find that the *Damage Reduction* condition increases voters’ preferred long-term investment level by $6 million (Model 1). This effect is statistically significant and meaningful in terms of magnitude. Compared to the control group, it translates into an increase of 13% over the baseline level of long-term investment. An important question is whether this effect is driven by the fact that respondents received no additional information about availability of responding to natural disasters by offering financial compensation for experienced losses. Two pieces of evidence speak against this interpretation. First, we find that the *Compensation* condition has no significant effect on investment preferences. Second, the damage reduction effect remains almost identical when assessed against the compensation treatment as an alternative control condition in which respondents did receive information about the possibility of providing relief aid. Models 2 and 3 in Table 2 report results from additional estimations that control for political and sociodemographic characteristics. As one would expect from a randomized experiment, our treatment effects remain unchanged.

**Table 2:** The Causal Effects of Policy Features on Preparedness Investment (in $ Million)

	(1)	(2)	(3)
Damage Reduction	5.25***	5.53***	5.24***
	(0.91)	(0.91)	(0.98)
Compensation	0.26	0.43	0.65
	(0.88)	(0.87)	(0.95)
Baseline: No Prime	44.43***	45.73***	49.75***
	(0.64)	(1.53)	(3.69)
Sociodemographics	No	Yes	Yes
Additional Controls	No	No	Yes
State Fixed Effects	No	No	Yes
Observations	2,618	2,618	2,271
R-squared	0.017	0.037	0.045

OLS coefficients shown with robust standard errors in parentheses. The dependent variable is the preferred level of investment in disaster preparedness. ***$p<0.01$, **$p<0.05$. Sociodemographic controls include a full set of income, age, education, gender, and race group indicators. Additional controls include partisanship, political trust, disaster affectedness, risk acceptance, patience, environmentalism, and responsibility beliefs

Which factors explain the impact of policy effectiveness on voters’ investment choices? To explore this question we perform several subgroup comparisons of the treatment effects. The subgroups are formed by variables that relate to the theoretical mechanisms laid out above. Online Appendix Table A.3 reports the wording of the survey items used to measure these variables and their descriptive statistics. We first evaluate whether the sensitivity to policy effectiveness can be explained by the environmentalism mechanism. In contrast to environmentalists, individuals who are less in favor of environmentalism may possess less knowledge about the effectiveness of long-term oriented policy instruments. As a result, the provision of such information should be more effective among non-environmentalists. To test this explanation we employ an environmental willingness-to-pay question item that has been used in previous research. We convert this measure into an indicator variable that equals 1 for individuals whose answer exceeded the median response and is zero otherwise. We use this indicator variable to partition our data and estimate the treatment effects separately for each of the two groups.

**Fig. 2:**
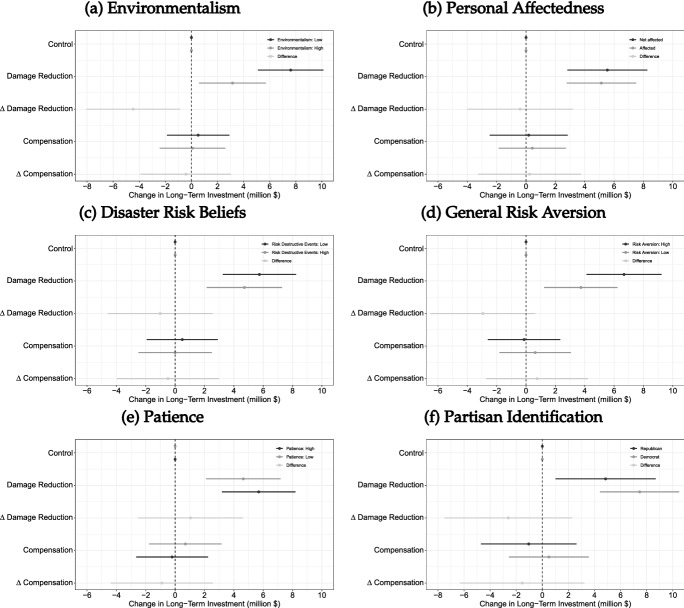
The causal effects of policy features on long-term investment by subgroups. *Note:* The plots show estimates of the causal effect of informational primes on long-term investment in disaster preparedness by subgroups. Estimates are based on a linear regression of *Long-Term Investment* on indicator variables with robust standard errors. $$\Delta$$ is the difference in the treatment effects. Horizontal lines indicate 95% robust confidence intervals. The baseline level of long-term investment in the control group is $44.5 million. $$N = 2618$$

Figure 2 shows the estimated treatment effects and 95% confidence intervals. We find that the damage reduction prime causes both environmentalists and non-environmentalists to allocate significantly more resources to funding preparedness (Fig. 2a). However, the treatment effect differs between the two groups. Environmentalists spend about $3.1 billion on preventive measures, while less environmentalist respondents spend $7.6 million on improving disaster preparedness. To test whether these estimates are significantly different from each other, we calculate the difference in the treatment effects along with the corresponding 95% confidence interval.^13^ Figure 2a reports this difference (which is − $4.5 million) and the corresponding 95% confidence interval. We find that the underlying effects are significantly different from each other. This indicates that knowledge about the economic features of long-term investment options changes policy preferences more strongly among less environmentalist individuals. We find no systematic differences when exploring the effect of the compensation treatment. This is again consistent with the view that the compensation condition corresponds to respondents’ baseline beliefs which is that the provision of relief aid is a feasible and effective response to extreme weather events.

Figure 2 also reports the treatment effects estimated separately for several subgroups that are of theoretical interest. We re-estimate the treatment effects using the median of each variable as the cutoff value.^14^ We find that the treatment effects are robust across a large set of dimensions such as personal affectedness, general risk attitudes, subjective disaster risk beliefs, personal time horizons, and partisan identification. Online Appendix Figure A.1 reports additional results for sociodemographic and political subgroups. These estimates further suggest that the causal effects of the damage reduction treatment are very similar across societal groups. Overall, the results are consistent with the argument that misperceptions about the long-term benefits of preparedness help explain mass preferences over disaster policy.

### Flood Control Dam Experiment: Design

A second source of opposition to investing in long-term prevention could be misperceptions about the relative costliness of this preventive policy option compared to the costs associated with the provision of disaster relief. To explore this argument we again pursue a research strategy that makes the financial burden of investing in disaster preparedness explicit by leveraging the randomly assigned construction costs in our flood control dam question which were either $2, $8, or $14 million. We use this vignette experiment to estimate the causal effects of costs by regressing support for the flood control dam on indicator variables that distinguish between *Medium Costs ($8 million)* and *Low Costs ($2 million)* while the *High Costs ($14 million)* condition serves as the control group.

### Flood Control Dam Experiment: Results

Model 1 in Table 3 reports the results. We find that respondents in the medium costs treatment are 1.7 percentage points more in favor of building the dam than individuals in the control group in which construction costs were set at $14 million although this effect is not statistically significant. The results indicate, however, that support for the flood control dam increases significantly by 4 percentage points if construction costs are set at $2 million. When interpreting these magnitudes it is useful to note that preventive investment is considerably more cost effective than relief spending. Although there will certainly exist variation in how much can be gained by investing in prevention instead of compensation, the low cost scenario in our dam experiment, in which a $2 million investment reduces future damage by $20 million seems within the possible range according to existing estimates. Models 2 and 3 in Table 3 add a large set of sociodemographic and attitudinal characteristics. As one would expect, the treatment effects remain unchanged.

**Table 3:** The Causal Effects of Construction Costs on Dam Support (in Percentage Points)

	(1)	(2)	(3)
Costs: Low ($2 Million)	4.76***	4.54***	4.27***
	(1.13)	(1.13)	(1.24)
Costs: Medium ($8 Million )	1.67	1.64	1.80
	(1.13)	(1.13)	(1.23)
Baseline Support (Costs: High, $14 Million )	62.88***	61.80***	61.15***
	(0.80)	(1.87)	(4.23)
Sociodemographics	No	Yes	Yes
Additional Controls	No	No	Yes
State Fixed Effects	No	No	Yes
Observations	2,618	2,618	2,271
R-squared	0.007	0.022	0.035

OLS coefficients shown with robust standard errors in parentheses. The dependent variable is the level of support for building a local flood control dam on a 1 to 10 scale that has been multiplied by 10 for ease of presentation. ***$p<0.01$, **$p<0.05$. Sociodemographic controls include a full set of income, age, education, gender, and race group indicators. Additional controls include partisanship, political trust, disaster affectedness, risk acceptance, patience, environmentalism, and responsibility beliefs

To explore heterogeneity in our treatment effects we separately estimate the results by theoretically meaningful subgroups. Online Appendix Figure A.2 (p. 9) reports these results. Overall, we find that the low cost treatment increases support among virtually all subgroups including voters with different partisan identifications. This indicates that Republicans—despite their well-documented preference for small government—are not fundamentally opposed to public investment in preparedness but rather that the willingness to back such investments is sensitive to their price. Second, previous research documents that individual attitudes toward environmental issues are increasingly structured along partisan lines (McCright et al., 2014). Our findings advance this research by revealing an important similarity: Republicans and Democrats tend to be equally sensitive to the price of improving disaster preparedness. The fact that the price sensitivity is quite similar across political groups also suggests that communication efforts to increase public approval of damage-reducing, long-term investment may be effective across partisan cleavages.

## Conclusion

The tendency of democracies to prioritize short-term responses over long-term solutions to important policy problems may result in considerable economic efficiency losses. If countries are underprepared for natural disasters such as floods, tornadoes, earthquakes, or pandemics, the resulting financial burden and human suffering  are ultimately imposed on future generations. Therefore, an important scientific task lies in identifying ways in which societies can craft sustainable solutions to major challenges such as climate change, economic crises, or infectious disease. The phenomenon of underinvestment in preparedness raises a range of theoretically interesting social science questions about the origins of mass preferences over long-term policymaking. To our knowledge, this is the first study to explore whether mass opposition to long-term preparedness investment reflects personal exposure to the underlying risks and misperceptions about the features of the available policy options.

First, we use stated and geo-coded measures of personal exposure to extreme weather events to explore the role of experiential learning. This argument holds that the willingness to devote public resources for preparedness instead of relief spending should depend on personal exposure to natural disasters. Higher levels of affectedness should increase support for investing in a local disaster preparedness project. However, the results suggest that neither subjective nor objective indicators of disaster exposure are systematically correlated with mass preferences over preparedness. This questions the learning-through-experience argument. Consequently, personal experience with major collective risks may be less consequential for the formation of policy preferences than previously thought.

Second, we devise two survey experiments to explore a potential explanation for the absence of a systematic link between exposure and policy preferences: misperceptions about the relative costs and benefits of long-term investment as opposed to the provision of disaster relief. If mass support for investing in preventive measures is muted because voters underestimate their effectiveness, the provision of such information should lead individuals to revise their policy preferences accordingly. The experimental results speak to these questions by offering three insights. First, greater reductions in long-term damage and lower costs increase the willingness to invest tax dollars in disaster preparedness. While the latter finding may simply reflect general cost aversion, the former result seems more consistent with the argument that misperceptions add to our understanding of myopia in disaster policy preferences. Second, we find that information about the possibility of providing relief aid in the aftermath of natural disasters fails to increase support for this policy option compared to the control group in which respondents receive no such prime. This result is informative since it indicates that the option of providing disaster relief captures the public’s baseline beliefs. Third, we find that the causal effects we document are quite stable across a large set of theoretically and politically important subgroups. Most notably, they are evident among both Republicans and Democrats. This homogeneity in the treatment effects implies that efforts to increase the public’s awareness of the economic features of preventive spending may have the potential to be effective among a large set of voters despite the strong partisan divide that characterizes attitudes toward climate and environmental policy.

Our study of whether experiential learning through personal exposure and misperceptions explain mass preferences over disaster policy ties into broader debates over the impact of economic shocks on political attitudes and the ability of democracies to adopt reforms that overcome myopic policymaking (Lindvall, 2017; Jacobs, 2011). Natural disasters constitute major economic shocks and previous research has documented that incumbents can realize large and durable electoral gains when providing timely disaster relief (Bechtel & Hainmueller, 2011; Gasper & Reeves, 2011; Healy & Malhotra, 2009). Our results demonstrate, however, that these electoral effects may not necessarily transform the willingness to back long-term investment in preparedness. The increasing frequency and intensity of extreme weather events may not only fail to generate broad public support for improving preparedness. It could even amplify the electoral incentives to engage in the more costly, short-term policy option of providing relief aid even though this approach could become financially unsustainable in the long-run.

Finally, underpreparedness paired with a strong reliance on compensatory measures also seems to characterize other issue areas such as how policymakers address public health threats originating from infectious disease. Countries are only beginning to fathom the costliness of the historically unprecedented relief spending bills passed by policymakers in response to the global COVID-19 pandemic. Future research may probe whether the public holds similar spending preferences in these issue areas and the extent to which exposure or misperceptions about the economic features of long-term investment strategies improves our understanding of the tendency to underprepare.

## Supplementary Information

Below is the link to the electronic supplementary material.

Electronic supplementary material 1 (PDF 166 kb)

## Footnotes

1 It is important to note that some climate adaptation measures may promise more immediate benefits, e.g., due to improved predictability and reliability of food supply and increased air quality.

2 “25 States Are at Risk of Serious Flooding This Spring, U.S. Forecast Says”, *New York Times*, March 21, 2019, https://nyti.ms/2Tn6A3l, accessed March 22, 2017; NOAA National Centers for Environmental Information (NCEI). “U.S. Billion-Dollar Weather and Climate Disasters (2017)”, accessed February 2, 2017, https://www.ncdc.noaa.gov/billions/; “Natural disasters cost $91 billion in 2018, according to federal report”, *CNBC*, https://www.cnbc.com/2019/02/06/natural-disasters-cost-91-billion-in-2018-federal-report.html. accessed September 22, 2019.

3 Specifically, average relief spending has been $6 billion per year while preparedness investment was $0.2 billion. These figures isolate spending programs that are directly related to disaster relief and disaster preparedness, see Bechtel and Mannino (2021) for more details.

4 A rival argument holds that having experienced natural disasters increases awareness of the destructiveness of these events which could lead individuals to support higher levels of relief as opposed to preparedness investment.

5 The survey was fielded in October 2016. Respondents were recruited by Respondi, an international survey firm.

6 In 2020, failure of two dams in Michigan allowed floodwater to enter a chemical complex that included a nuclear reactor and endangered a toxic-cleanup site. The damage to homes and businesses has been estimated at $175 million, see https://www.nytimes.com/2020/05/20/climate/michigan-dam-dow-chemical-superfund.html.

7 The raw data is accessible at http://hvri.geog.sc.edu/SHELDUS. If a disaster affects multiple counties, the total damage and associated fatalities are distributed equally across these counties.

8 Note that since our survey contained experimental items, which we report in detail further below, these descriptives are based on respondents’ allocation decisions in the control condition, i.e., where respondents received no additional information in the preparedness investment question and received a low costs prime in the dam support question.

9 This analysis utilizes the entire sample ($N=2618$) since the experimental manipulations were fully randomized. As one would expect, the results are very similar when controlling for treatment conditions and when re-estimating the main models by experimental condition (Online Appendix Table A.4).

10 For example, one could employ more subtle informational prompts. The associated risk would be that any null results could either be due to the treatments not having been explicit enough or individuals being indeed insensitive to disaster policy features.

11 The economic damage caused by Hurricane Katrina has been estimated at $108 billion (Knapp et al., 2012) and the efficiency gains from investing in preparedness could have reduced this quantity by a factor of about 15 (Healy & Malhotra, 2009). “Hurricane Katrina, August 23–30, 2005”, *Goddard Earth Science Data and Information Services Center*,https://disc.gsfc.nasa.gov/hurricane/additional/science-focus/HurricaneKatrina2005.shtml, http://www.nhc.noaa.gov/data/tcr/AL122005_Katrina.pdf.

12 The robustness section reports results from tobit estimations that account for the bounded nature of our outcome variable. The results remain identical.

13 This step is necessary since visually inspecting overlap between the corresponding confidence intervals is typically insufficient to determine whether the difference in two estimated effects is statistically significant (Cumming & Finch, 2005).

14 Online Appendix Table A.3 reports the exact question wording and definitions for these variables.
