# Supplementary material for: Ready When the Big One Comes? Natural Disasters and Mass Support for Preparedness Investment
Source: Polit Behav. 2021 Aug 21;45(3):1045–70. doi: 10.1007/s11109-021-09738-2 (PMC8380007; doi:10.1007/s11109-021-09738-2)
Supplement: Supplementary file 1 — Electronic supplementary material 1 (PDF 166 kb) [file 11109_2021_9738_MOESM1_ESM.pdf]

Ready When the Big One Comes? Natural Disasters and Mass Support for Preparedness Investment

Online Appendix

A Appendix Tables

Table A.1: Distribution of Socidemographics in the Target Population, Weighted Sample, Raw Sample (in %)

	Population	Weighted Sample	Raw Sample
Age: 18-24	12.6	12.7	16.8
Age: 25-44	34.2	34.2	35
Age: 45-64	33.9	33.8	30.6
Age: 65+	19.3	19.3	17.5
Gender: Male	48.7	48.7	47.3
Gender: Female	51.3	51.3	52.7
Education: Less than High School	12.2	12.2	10.6
Education: High School Degree	29.6	29.6	28.3
Education: Some College	28.4	28.5	31.6
Education: Bachelor's Degree	19.2	19.2	18
Education: Advanced Degree	10.6	10.5	11.3

Note: The table reports the distribution of socio-demographic characteristics in the population, the weighted sample, and the unweighted (raw) sample. N = 2,618. The population margins are based on the 2016 Current Population Survey, see http://www.census.gov/cps/data/cpstablecreator.html.

Table A.2: Descriptive Statistics: Self-reported Affectedness

**Variable**	**Myself**	**Other Household Members**
Not affected	63%	53%
Closed/restricted roads or (partial) shutdown of public transportation	37%	28%
Shutdown of school/university	23%	20%
Communication problems	26%	20%
Restricted access to food or water	18%	15%
Evacuation	14%	13%
Financial loss	19%	15%
Injury	13%	12%

Note: The table reports the share of respondents affected by at least one disaster. The corresponding question wording is: "In the past years, natural disasters such as tornadoes, floods, earthquakes, droughts, and other adverse weather events have affected several regions in the United States. We are interested in learning about whether you have experienced or not experienced one or more natural disasters. Please indicate below whether you or members of your household were affected by any of the following events as a consequence of a natural disaster in the past ten years (since 2006). If you or members of your household were not affected please select 'Not affected'. If you were affected, please select all that apply." N = 2,618.

Table A.3: Variable Definitions and Descriptives

Variable and Question Wording	Mean	Median	Std. Dev.	Min.	Max.
**Risk Aversion**<br>"Are you generally trying to avoid taking risks, or are you willing to take risks?"<br>Scale: 0 (Completely unwilling to take risks) - 10 (Very willing to take risks)	4.1	3.0	2.7	0	10
**Patience (Time Preference)**<br>"Are you generally willing to give up something today in order to benefit from that in the future or are you not willing to do so?"<br>Scale: 0 (Completely unwilling to give up something today) - 10 (Very willing to give up something today)	5.9	6.0	2.5	0	10
**Trust in Government**<br>"On a scale from 1 to 10, where 1 means that you do not trust the government, and 10 means that you trust the government, where would you put yourself?"<br>Scale: 1 (I do not trust the government) - 10 (I trust the government)	4.5	4.0	2.5	1	10
**Preparedness Responsibility**<br>"When thinking about preparing for a natural disaster, where do you place your view on the following scale?"<br>Scale: 1 (Each person should take responsibility for his/her own preparedness) - 10 (The government should take responsibility for disaster preparedness)	5.4	5.0	2.6	1	10
**Risk of Destructive Events**<br>"How unlikely or likely do you think is it that the county you live in will be affected by natural disasters such as earthquakes, tornadoes, droughts, wildfire, or other adverse weather events in 2017? Please give your answer on the following scale, where 0 means it is very unlikely that you will be affected and 10 means it is very likely that you will be affected."<br>Scale: 0 (Very unlikely) - 10 (Very likely)	5.1	5.0	3.0	0	10
**Environmentalism**<br>"How much of your monthly income would you be willing to invest into reducing greenhouse gas emissions (for example, buying energy efficient electric appliances, installing heat insulation in your home, buying electric power produced from renewable energy sources, buying locally produced food)? Please indicate the amount on a scale from 0 to 100, with 0 meaning 'nothing at all' and 100 meaning 'my whole income'."<br>Scale: 0 (Nothing at all) - 100 (My whole income)	28.5	19.0	27.7	0	100

Note: N = 2,618.

Table A.4: The Correlates of Support for Disaster Preparedness Investment, Treatment Indicators Included and by Experimental Condition

Outcome	Preparedness Investment (%)								Dam Support (1-10)x10							
Treatment Condition:	Pooled	Control	Damage	Compensation	Pooled	Control	Damage	Compensation	Pooled	Costs: Low	Costs: Medium	Costs: High	Pooled	Costs: Low	Costs: Medium	Costs: High
Model:	(1)	(2)	(3)	(4)	(5)	(6)	(7)	(8)	(9)	(10)	(11)	(12)	(13)	(14)	(15)	(16)
Personal Affectedness: Medium	0.59	1.83	-0.10	0.17					0.63	-0.99	0.38	2.69				
	(0.99)	(1.72)	(1.74)	(1.76)					(1.18)	(2.03)	(2.11)	(2.09)				
Personal Affectedness: High	0.08	-0.19	-2.09	2.12					-2.25	-5.58**	-2.13	-0.14				
	(1.10)	(1.91)	(2.04)	(1.96)					(1.43)	(2.50)	(2.49)	(2.55)				
Disaster Risk: Medium	-0.38	1.62	-1.14	-0.71					-0.48	-1.33	-2.50	0.72				
	(0.98)	(1.77)	(1.64)	(1.83)					(1.22)	(2.22)	(2.10)	(2.16)				
Disaster Risk: High	-0.24	-0.86	0.67	-0.09					2.02	1.30	3.01	0.39				
	(1.07)	(1.85)	(1.99)	(1.87)					(1.32)	(2.32)	(2.32)	(2.38)				
Injuries: Medium					-0.55	-0.10	-1.20	-0.43					0.42	2.78	-1.59	-0.69
					(1.02)	(1.76)	(1.71)	(1.82)					(1.30)	(2.30)	(2.29)	(2.33)
Injuries: High					-0.50	1.11	-2.38	-1.45					-2.21	0.44	-6.33**	-1.20
					(1.27)	(2.18)	(2.31)	(2.29)					(1.58)	(2.79)	(2.82)	(2.62)
Economic Damage: Medium					1.19	1.76	2.40	0.24					0.70	-2.99	2.79	3.77
					(1.08)	(1.97)	(1.81)	(1.89)					(1.32)	(2.52)	(2.25)	(2.32)
Economic Damage: High					-0.12	0.72	-1.09	0.76					-0.19	0.66	1.76	-3.00
					(1.28)	(2.33)	(2.15)	(2.29)					(1.62)	(2.78)	(3.01)	(2.77)
Patience: Medium	0.46	-0.96	0.77	0.69	0.79	0.38	0.90	0.33	0.94	2.34	0.09	-1.02	0.69	2.00	0.90	-1.95
	(1.08)	(1.85)	(1.87)	(2.09)	(1.07)	(1.86)	(1.83)	(2.09)	(1.35)	(2.43)	(2.33)	(2.52)	(1.34)	(2.42)	(2.35)	(2.45)
Patience: High	2.16*	2.25	1.77	2.95	2.27**	2.36	2.07	2.56	6.82***	7.33***	8.35***	4.04	6.41***	6.82***	8.44***	3.78
	(1.11)	(1.99)	(1.82)	(2.06)	(1.10)	(1.96)	(1.79)	(2.07)	(1.42)	(2.63)	(2.45)	(2.56)	(1.41)	(2.63)	(2.42)	(2.53)
Risk Acceptance: Medium	0.78	2.33	1.12	-0.14	1.02	2.10	1.04	1.20	0.26	1.04	-1.91	2.85	0.57	1.29	-2.48	3.34
	(1.39)	(2.57)	(2.31)	(2.29)	(1.36)	(2.54)	(2.27)	(2.30)	(1.73)	(2.93)	(3.17)	(2.92)	(1.73)	(2.96)	(3.18)	(2.82)
Risk Acceptance: High	0.54	2.42	1.83	-1.13	0.73	1.61	1.56	0.72	1.67	2.08	-0.26	4.38	1.85	2.38	-1.26	4.64
	(1.47)	(2.63)	(2.49)	(2.54)	(1.44)	(2.61)	(2.41)	(2.52)	(1.87)	(3.15)	(3.41)	(3.25)	(1.85)	(3.18)	(3.35)	(3.17)
Environmentalism: Medium	1.53	4.11**	0.81	0.85	1.56	3.40**	0.82	1.23	3.27***	4.44**	5.00**	0.74	3.09***	3.78*	4.30*	1.94
	(1.00)	(1.75)	(1.74)	(1.83)	(0.98)	(1.72)	(1.66)	(1.83)	(1.21)	(2.10)	(2.19)	(2.18)	(1.20)	(2.09)	(2.19)	(2.16)
Environmentalism: High	4.06***	6.56***	5.12**	1.67	4.04***	5.75***	4.91**	2.28	-0.96	-0.84	4.04	-5.62**	-1.24	-3.05	3.91	-4.44*
	(1.18)	(2.18)	(2.08)	(2.07)	(1.14)	(2.10)	(1.97)	(1.99)	(1.41)	(2.63)	(2.46)	(2.47)	(1.40)	(2.62)	(2.42)	(2.40)
Responsibility Beliefs: Medium	1.44	-1.66	1.12	6.50***	1.55	-2.14	1.61	6.28***	0.26	-1.06	5.10*	-3.98	0.92	0.51	4.70*	-3.53
	(1.17)	(2.10)	(1.86)	(2.25)	(1.14)	(2.11)	(1.79)	(2.23)	(1.47)	(2.62)	(2.63)	(2.57)	(1.46)	(2.63)	(2.63)	(2.51)
Responsibility Beliefs: High	1.65	-0.69	1.97	4.62**	1.97*	-0.98	2.84	5.04**	0.26	-0.61	2.77	-1.47	0.65	0.74	2.35	-1.56
	(1.13)	(1.98)	(1.88)	(2.13)	(1.11)	(2.01)	(1.81)	(2.12)	(1.40)	(2.47)	(2.53)	(2.43)	(1.39)	(2.47)	(2.53)	(2.38)
Political Trust: Medium	-1.21	0.40	-4.26**	0.04	-1.21	0.78	-4.72**	-0.01	-0.58	-2.50	1.42	0.52	-0.34	-1.88	1.12	0.01
	(1.15)	(2.04)	(1.93)	(2.09)	(1.15)	(2.04)	(1.90)	(2.11)	(1.43)	(2.43)	(2.68)	(2.54)	(1.43)	(2.45)	(2.66)	(2.46)
Political Trust: High	-0.26	1.19	-0.19	-1.84	-0.46	1.14	-0.95	-1.48	-0.04	-4.46*	3.29	1.97	0.20	-3.67	2.94	0.97
	(1.25)	(2.20)	(2.18)	(2.28)	(1.25)	(2.20)	(2.12)	(2.31)	(1.59)	(2.58)	(2.97)	(2.82)	(1.58)	(2.57)	(2.90)	(2.77)
Partisanship: Republican	0.00	2.07	0.15	-1.77	0.49	3.72*	0.06	-1.26	-5.35***	-5.80**	-3.52	-7.17**	-4.91***	-4.34	-3.25	-6.67**
	(1.20)	(2.14)	(2.12)	(2.13)	(1.18)	(2.07)	(2.12)	(2.10)	(1.55)	(2.65)	(2.70)	(2.89)	(1.56)	(2.74)	(2.69)	(2.83)
Partisanship: Other	0.02	1.21	1.53	-1.67	0.17	1.63	1.53	-1.61	-5.54***	-5.75***	-3.87*	-6.35***	-5.37***	-4.91**	-4.17**	-5.74***
	(0.96)	(1.77)	(1.67)	(1.70)	(0.95)	(1.75)	(1.66)	(1.70)	(1.21)	(2.15)	(2.05)	(2.20)	(1.21)	(2.22)	(2.03)	(2.15)
Income: Medium	-0.55	-0.97	0.10	-0.85	-0.39	-0.00	0.01	-1.15	1.31	0.92	2.71	0.48	2.04	1.74	3.69	1.47
	(1.02)	(1.84)	(1.79)	(1.81)	(1.00)	(1.83)	(1.72)	(1.78)	(1.27)	(2.33)	(2.28)	(2.16)	(1.28)	(2.37)	(2.26)	(2.15)
Income: High	0.46	-0.08	-1.03	2.91	0.41	-0.07	-0.98	2.47	1.93	1.79	2.17	0.66	2.66**	2.20	3.02	2.96
	(1.09)	(2.01)	(1.83)	(1.98)	(1.09)	(1.97)	(1.81)	(2.02)	(1.33)	(2.46)	(2.22)	(2.37)	(1.32)	(2.49)	(2.28)	(2.28)
Age: 25-44	-0.84	-1.31	-3.63	3.74	-0.11	-0.05	-4.03*	4.84**	-3.88**	-5.69*	-6.32**	-0.10	-4.71***	-5.59*	-6.98***	-2.15
	(1.31)	(2.27)	(2.28)	(2.38)	(1.31)	(2.30)	(2.25)	(2.36)	(1.62)	(3.01)	(2.75)	(2.88)	(1.59)	(3.03)	(2.69)	(2.82)
Age: 45-64	-2.14	-3.83	-4.62*	3.35	-1.67	-2.59	-4.73**	3.96	-0.80	-1.69	-1.20	0.23	-1.43	-1.93	-2.22	-1.24
	(1.38)	(2.41)	(2.38)	(2.45)	(1.35)	(2.39)	(2.35)	(2.42)	(1.67)	(2.99)	(2.84)	(3.03)	(1.65)	(3.02)	(2.80)	(2.90)
Age: 65 or more	-3.44**	-4.25	-7.33***	3.04	-3.04**	-3.14	-7.81***	3.66	0.64	-0.05	3.23	-1.63	-0.03	-0.14	2.11	-2.99
	(1.56)	(2.83)	(2.67)	(2.79)	(1.53)	(2.80)	(2.63)	(2.74)	(1.87)	(3.32)	(3.34)	(3.32)	(1.84)	(3.37)	(3.27)	(3.21)
Education: High School	-1.57	-0.31	0.41	-4.64*	-1.24	0.43	-0.68	-3.51	1.95	0.78	2.80	2.13	0.88	-0.94	1.26	2.33
	(1.53)	(2.95)	(2.50)	(2.48)	(1.49)	(2.90)	(2.37)	(2.46)	(1.92)	(3.73)	(3.27)	(3.30)	(1.89)	(3.81)	(3.29)	(3.11)
Education: Some College	-1.11	-0.62	-0.41	-1.81	-0.84	-0.20	-1.61	-0.52	0.12	2.10	0.23	-1.70	-1.15	-0.48	-0.87	-1.96
	(1.56)	(2.95)	(2.52)	(2.61)	(1.53)	(2.93)	(2.39)	(2.59)	(1.97)	(3.74)	(3.41)	(3.36)	(1.93)	(3.81)	(3.42)	(3.17)
Education: BA	0.71	3.57	3.28	-4.39	1.08	3.73	2.49	-3.54	1.37	7.17*	1.36	-2.68	-0.13	4.89	-0.21	-3.41
	(1.69)	(3.27)	(2.68)	(2.97)	(1.66)	(3.23)	(2.53)	(2.92)	(2.15)	(4.03)	(3.82)	(3.64)	(2.13)	(4.18)	(3.85)	(3.49)
Education: Advanced Degree	2.93	4.29	5.70*	-1.70	2.96	4.31	4.72	-0.99	2.16	1.89	5.29	-0.39	1.02	-0.31	3.30	-0.38
	(1.89)	(3.31)	(3.10)	(3.49)	(1.89)	(3.33)	(3.05)	(3.54)	(2.53)	(4.83)	(4.48)	(4.38)	(2.52)	(4.98)	(4.55)	(4.21)
Female	-0.69	1.07	-0.84	-2.52	-0.77	0.40	-0.86	-2.17	-0.34	0.56	0.75	-1.19	-0.22	1.36	0.34	-0.86
	(0.90)	(1.64)	(1.52)	(1.59)	(0.89)	(1.67)	(1.48)	(1.60)	(1.06)	(1.86)	(1.85)	(1.87)	(1.07)	(1.92)	(1.89)	(1.84)
Race: African American	0.49	-0.22	2.51	1.26	1.07	-0.06	3.34	1.84	-2.81	-9.30**	-0.21	-1.40	-2.65	-8.72**	-0.56	0.24
	(1.56)	(2.77)	(2.81)	(2.76)	(1.51)	(2.69)	(2.64)	(2.68)	(2.04)	(3.64)	(3.20)	(3.57)	(2.01)	(3.89)	(3.24)	(3.29)
Race: Hispanic	0.39	-2.39	2.21	1.87	0.34	-3.50	3.54	1.19	0.88	-0.46	2.71	0.93	0.97	-0.25	2.32	1.59
	(1.68)	(2.60)	(3.29)	(2.95)	(1.69)	(2.74)	(3.20)	(2.98)	(2.25)	(3.91)	(3.76)	(3.92)	(2.23)	(3.93)	(3.68)	(3.88)
Race: Other	0.59	-0.91	1.26	2.04	0.25	-0.74	1.15	0.73	-3.54*	-0.90	-2.32	-7.71**	-3.59*	-1.30	-3.75	-6.96*
	(1.50)	(2.21)	(2.57)	(2.90)	(1.53)	(2.29)	(2.58)	(3.15)	(2.03)	(3.57)	(3.54)	(3.72)	(2.01)	(3.38)	(3.50)	(3.64)
Constant	46.25***	29.29***	54.56***	35.73***	47.35***	33.03***	44.37***	32.48***	68.03***	71.05***	60.37***	64.53***	60.60***	67.81***	65.86***	64.63***
	(4.19)	(8.34)	(6.93)	(6.66)	(3.98)	(7.16)	(6.68)	(6.58)	(4.93)	(8.43)	(11.07)	(9.62)	(5.41)	(8.45)	(10.48)	(9.47)
Treatment Indicators	Yes	Yes	Yes	Yes	Yes	Yes	Yes	Yes	Yes	Yes	Yes	Yes	Yes	Yes	Yes	Yes
State Fixed Effects	Yes	Yes	Yes	Yes	Yes	Yes	Yes	Yes	Yes	Yes	Yes	Yes	Yes	Yes	Yes	Yes
Observations	2,192	744	721	727	2,241	766	730	745	2,192	731	731	730	2,241	738	751	752
R-squared	0.060	0.079	0.113	0.080	0.057	0.066	0.118	0.072	0.080	0.120	0.130	0.111	0.074	0.109	0.124	0.113

Note: OLS coefficients shown with robust standard errors in parentheses (*** $p < 0.01$, ** $p < 0.05$, * $p < 0.1$).

Table A.5: The Correlates of Support for Disaster Preparedness Investment, Binary Affectedness Indicator

Model:	Preparedness Investment (%)	Dam Support (1-10)x10
Personal Affectedness: None (Reference group)		
Personal Affectedness: Some	0.37	-0.47
	(0.87)	(1.06)
Disaster Risk: Medium	-0.36	-0.50
	(0.98)	(1.23)
Disaster Risk: High	-0.22	2.19*
	(1.07)	(1.33)
Patience: Medium	0.44	0.82
	(1.08)	(1.35)
Patience: High	2.16*	6.82***
	(1.11)	(1.43)
Risk Acceptance: Medium	0.82	0.48
	(1.39)	(1.73)
Risk Acceptance: High	0.57	1.73
	(1.46)	(1.87)
Environmentalism: Medium	1.53	3.23***
	(1.00)	(1.22)
Environmentalism: High	3.99***	-1.29
	(1.17)	(1.41)
Responsibility Beliefs: Medium	1.47	0.52
	(1.16)	(1.47)
Responsibility Beliefs: High	1.65	0.39
	(1.13)	(1.40)
Political Trust: Medium	-1.21	-0.75
	(1.15)	(1.44)
Political Trust: High	-0.27	-0.10
	(1.25)	(1.59)
Partisanship: Republican	0.02	-5.25***
	(1.19)	(1.56)
Partisanship: Other	0.04	-5.51***
	(0.96)	(1.21)
Constant	46.19***	69.34***
	(4.19)	(4.96)
Sociodemographics	Yes	Yes
Treatment Indicators	Yes	Yes
State Fixed Effects	Yes	Yes
Observations	2,192	2,192
R-squared	0.060	0.073

Note: OLS coefficients shown with robust standard errors in parentheses (*** $p < 0.01$, ** $p < 0.05$, * $p < 0.1$). Sociodemographic controls include a full set of income, age, education, gender, and race group indicators.

Table A.6: The Correlates of Support for Long-Term Disaster Policy Investment (Tobit Estimates)

Outcome:	Long-Term Investment (in $ Million)						Dam Support (1-10)x10					
Model:	(1)	(2)	(3)	(4)	(5)	(6)	(7)	(8)	(9)	(10)	(11)	(12)
Personal Affectedness: Medium	0.68				0.44		1.67				0.74	
	(0.90)				(1.00)		(1.27)				(1.37)	
Personal Affectedness: High	0.57				-0.08		-1.02				-2.08	
	(1.00)				(1.13)		(1.54)				(1.69)	
Disaster Risk: Medium	0.07				-0.39		-0.72				-0.57	
	(0.93)				(0.99)		(1.33)				(1.41)	
Disaster Risk: High	0.23				-0.25		3.21**				2.44	
	(0.99)				(1.09)		(1.45)				(1.57)	
Injuries: Medium		-0.77				-0.82		0.96				0.15
		(0.90)				(1.03)		(1.34)				(1.52)
Injuries: High		-0.94				-0.75		-1.40				-2.93
		(1.12)				(1.28)		(1.64)				(1.85)
Economic Damage: Medium		0.90				1.46		1.10				1.25
		(0.94)				(1.10)		(1.34)				(1.53)
Economic Damage: High		0.22				0.02		0.69				0.49
		(1.10)				(1.29)		(1.73)				(1.92)
Patience: Medium			0.78		0.59	0.91			1.13		0.99	0.76
			(1.09)		(1.10)	(1.09)			(1.52)		(1.53)	(1.53)
Patience: High			2.16*		2.36**	2.48**			8.21***		8.30***	7.88***
			(1.11)		(1.13)	(1.12)			(1.64)		(1.66)	(1.65)
Risk Acceptance: Medium			1.09		0.76	0.92			0.22		-0.24	0.12
			(1.39)		(1.43)	(1.39)			(2.09)		(2.10)	(2.10)
Risk Acceptance: High			0.95		0.37	0.51			1.91		1.72	1.93
			(1.47)		(1.50)	(1.47)			(2.23)		(2.27)	(2.26)
Environmentalism: Medium				1.30	1.40	1.45				3.63***	3.59**	3.43**
				(0.90)	(1.02)	(0.99)				(1.29)	(1.42)	(1.40)
Environmentalism: High				3.19***	4.13***	4.11***				-0.91	-0.87	-1.03
				(1.06)	(1.21)	(1.16)				(1.48)	(1.66)	(1.64)
Responsibility Beliefs: Medium				1.94*	1.52	1.68				0.41	0.91	1.58
				(1.05)	(1.18)	(1.15)				(1.54)	(1.71)	(1.71)
Responsibility Beliefs: High				2.04**	1.71	2.10*				0.34	0.76	1.16
				(1.02)	(1.14)	(1.11)				(1.49)	(1.63)	(1.63)
Political Trust: Medium				-1.01	-1.03	-1.00				-1.71	-1.39	-1.05
				(1.05)	(1.17)	(1.17)				(1.51)	(1.69)	(1.68)
Political Trust: High				-0.71	-0.21	-0.42				0.26	-0.47	-0.16
				(1.16)	(1.28)	(1.27)				(1.72)	(1.89)	(1.88)
Partisanship: Republican			0.16	0.09	0.11	0.58			-6.60***	-4.88***	-6.40***	-5.83***
			(1.13)	(1.10)	(1.21)	(1.20)			(1.76)	(1.66)	(1.83)	(1.84)
Partisanship: Other			-0.14	0.07	-0.07	0.14			-6.72***	-6.95***	-6.50***	-6.30***
			(0.95)	(0.87)	(0.98)	(0.96)			(1.38)	(1.31)	(1.44)	(1.43)
Income: Medium	-0.68	-0.39	-0.81	-0.54	-0.56	-0.40	1.83	3.13**	1.61	1.85	0.97	1.96
	(0.96)	(0.96)	(1.02)	(0.94)	(1.03)	(1.02)	(1.37)	(1.38)	(1.47)	(1.36)	(1.47)	(1.48)
Income: High	0.61	0.83	0.02	0.55	0.37	0.26	3.20**	4.29***	3.25**	3.42**	2.35	3.29**
	(1.03)	(1.03)	(1.09)	(1.01)	(1.11)	(1.11)	(1.46)	(1.46)	(1.52)	(1.42)	(1.55)	(1.55)
Age: 25-44	-0.44	0.07	0.26	0.05	-0.86	-0.06	-4.92***	-5.81***	-6.28***	-5.63***	-5.52***	-6.45***
	(1.21)	(1.23)	(1.31)	(1.20)	(1.32)	(1.33)	(1.78)	(1.77)	(1.88)	(1.74)	(1.89)	(1.87)
Age: 45-64	-2.77**	-2.35*	-2.13	-1.75	-1.95	-1.48	-0.24	-1.35	-1.62	-1.28	-1.60	-2.28
	(1.25)	(1.25)	(1.35)	(1.24)	(1.39)	(1.37)	(1.81)	(1.78)	(1.92)	(1.78)	(1.98)	(1.95)
Age: 65 or more	-4.22***	-3.80***	-3.67**	-2.87**	-3.31**	-2.85*	0.98	0.14	0.30	-0.44	0.19	-0.56
	(1.41)	(1.39)	(1.50)	(1.40)	(1.58)	(1.54)	(2.05)	(1.99)	(2.14)	(2.02)	(2.23)	(2.19)
Education: High School	-1.10	-0.91	-1.08	-0.68	-1.49	-1.12	2.59	1.58	1.89	1.34	2.75	1.43
	(1.48)	(1.46)	(1.53)	(1.43)	(1.59)	(1.55)	(2.14)	(2.12)	(2.23)	(2.06)	(2.26)	(2.24)
Education: Some College	-0.71	-0.60	-0.59	-0.16	-1.10	-0.88	1.81	0.35	-0.49	0.01	0.17	-1.35
	(1.49)	(1.47)	(1.57)	(1.44)	(1.62)	(1.59)	(2.18)	(2.14)	(2.29)	(2.09)	(2.31)	(2.28)
Education: BA	0.92	1.54	1.24	1.58	0.66	1.11	2.28	0.90	0.36	0.29	1.61	-0.24
	(1.59)	(1.57)	(1.67)	(1.55)	(1.74)	(1.70)	(2.38)	(2.36)	(2.52)	(2.31)	(2.53)	(2.52)
Education: Advanced Degree	3.81**	3.54*	3.52*	3.89**	2.96	2.90	2.89	1.50	1.78	0.51	2.87	1.38
	(1.80)	(1.81)	(1.91)	(1.78)	(1.94)	(1.94)	(2.80)	(2.82)	(2.99)	(2.73)	(3.02)	(3.02)
Female	-1.30	-1.29	-0.80	-1.37*	-0.83	-0.85	-0.19	0.12	-0.16	-0.07	-0.35	-0.25
	(0.84)	(0.84)	(0.90)	(0.82)	(0.91)	(0.91)	(1.18)	(1.19)	(1.24)	(1.15)	(1.25)	(1.26)
Race: African American	1.12	1.49	1.41	0.86	0.40	1.02	-2.35	-2.47	-3.83	-3.98*	-3.23	-3.16
	(1.50)	(1.47)	(1.54)	(1.45)	(1.59)	(1.54)	(2.24)	(2.22)	(2.33)	(2.21)	(2.38)	(2.35)
Race: Hispanic	1.50	0.97	0.97	0.65	0.76	0.64	1.46	1.14	0.37	1.08	1.23	1.14
	(1.63)	(1.65)	(1.72)	(1.60)	(1.72)	(1.73)	(2.53)	(2.50)	(2.68)	(2.43)	(2.69)	(2.68)
Race: Other	1.64	1.06	0.35	1.12	0.55	0.16	-4.15*	-4.38*	-4.99**	-4.77**	-4.17*	-4.27*
	(1.43)	(1.48)	(1.56)	(1.44)	(1.54)	(1.56)	(2.24)	(2.24)	(2.40)	(2.21)	(2.43)	(2.39)
Constant	47.99***	47.86***	46.19***	45.01***	43.93***	43.52***	63.78***	65.34***	59.91***	70.75***	58.42***	60.79***
	(1.67)	(1.54)	(3.74)	(2.10)	(4.01)	(4.03)	(2.41)	(2.21)	(4.67)	(3.02)	(5.15)	(5.22)
State Fixed Effects					Yes	Yes					Yes	Yes
Observations	2,517	2,543	2,271	2,618	2,192	2,241	2,517	2,543	2,271	2,618	2,192	2,241

Note: Tobit coefficients shown with robust standard errors in parentheses (*** $p < 0.01$, ** $p < 0.05$, * $p < 0.1$).

B Appendix Figures

Figure A.1: The Causal Effects of Policy Features on Preparedness Investment by Subgroups

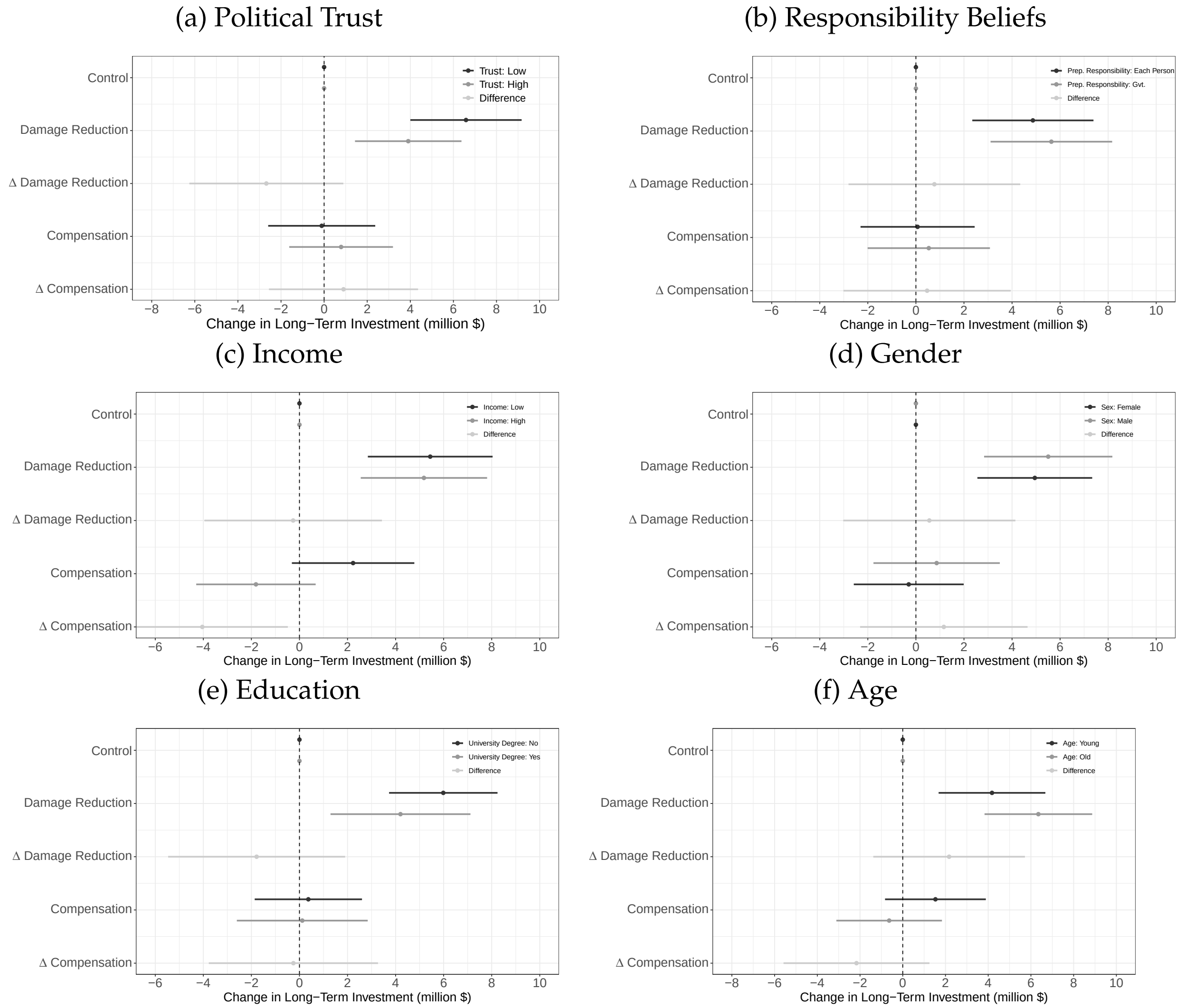

Note: The plots report estimates of the causal effects of disaster policy features on preparedness spending by subgroups. Estimates are based on a linear regression of *Long-Term Investment* on treatment indicators. Δ is the difference in the treatment effects. Horizontal lines indicate 95% robust confidence intervals. Baseline level of long-term investment in the control group is \$44.5 million. $N = 2,618$.

Figure A.2: The Causal Effects of Construction Costs on Support for Flood Control Dams by Subgroups

(a) Environmentalism

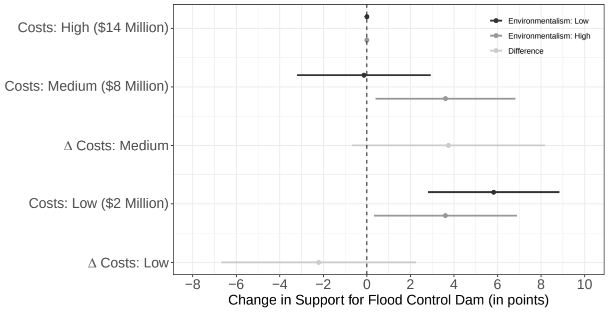

(b) Personal Affectedness

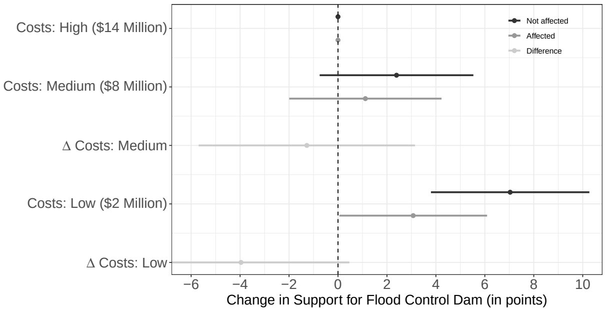

(c) Disaster Risk Beliefs

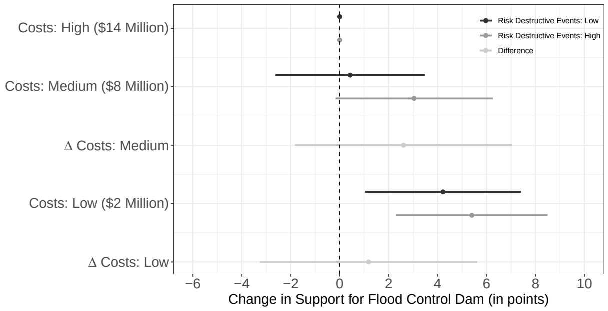

(d) General Risk Aversion

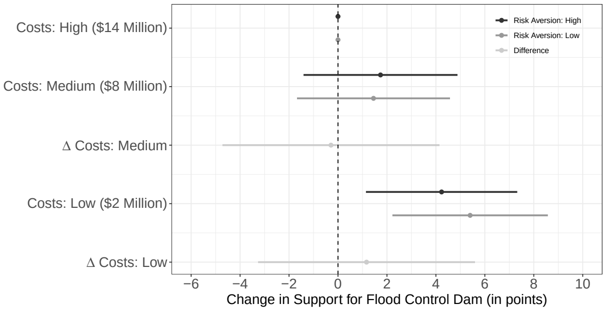

(e) Patience

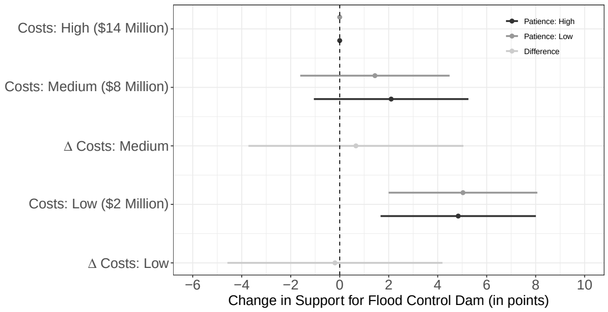

(f) Partisan Identification

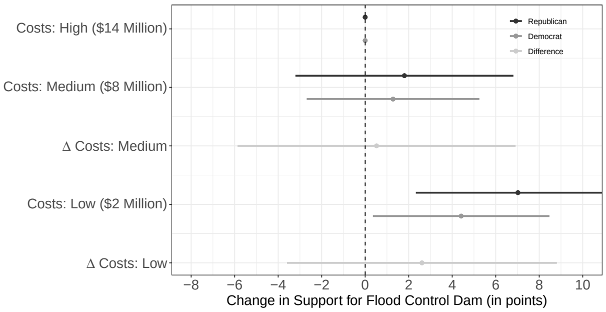

Note: The plots report estimates of the causal effects of disaster preparedness costs on support for a flood control dam by subgroups. Estimates are based on a linear regression of *Dam Support* on treatment indicators. δ is the difference in the treatment effects. Horizontal lines indicate 95% robust confidence intervals. Baseline level of support is 53 (scale: 1-100). $N = 2,618$.
